# Malignant Pleural Mesothelioma CT Imaging: How to Measure It Correctly?

**DOI:** 10.1177/10732748241301901

**Published:** 2025-03-16

**Authors:** Carmine Picone, Annamaria Porto, Roberta Fusco, Vincenza Granata, Maria Chiara Brunese, Agnese Montanino, Giovanna Esposito, Raffaele Costanzo, Anna Manzo, Vincenzo Sforza, Claudia Sandomenico, Giuliano Palumbo, Edoardo Mercadante, Alessandro Ottaiano, Gianfranco Vallone, Ferdinando Caranci, Raffaella Mormile, Alessandro Morabito, Antonella Petrillo

**Affiliations:** 1 Radiology Division, Istituto Nazionale Tumori IRCCS Fondazione G. Pascale, Napoli, Italy; 2 18960 Radiology Division, Università degli Studi del Molise, Campobasso, Italy; 3 Department of Thoracic Pulmonary Oncology, Istituto Nazionale Tumori IRCCS Fondazione G. Pascale, Napoli, Italy; 4 Department of Thoracic Pulmonary Surgery, Istituto Nazionale Tumori IRCCS Fondazione G. Pascale, Napoli, Italy; 5 653993 Department of Innovative Therapies of Abdominal Cancer, Istituto Nazionale Tumori IRCCS Fondazione G. Pascale, Napoli, Italy; 6 220495 Radiology Division, Università Degli Studi Della Campania “Luigi Vanvitelli”, Napoli, Italy; 7 220495 Pediatric Division, San Giusppe Moscati Hospital, Aversa, Italy

**Keywords:** malignant pleural mesothelioma, computed tomography, RECIST criteria

## Abstract

**Background:** Malignant pleural mesothelioma is the most common primary tumor of the pleura. The unique growth pattern of malignant pleural mesothelioma makes it difficult to apply the Response Evaluation Criteria for Solid Tumors (RECIST). Hence the need to use modified RECIST (mRECIST) criteria, as they better fit the unique growth pattern of malignant pleural mesothelioma. The thickness of the tumor perpendicular to the chest wall or mediastinum is measured at 2 points at 3 separate levels at least 1 cm apart on chest CT scans, and summed to obtain a one-dimensional pleural measurement. The same criterion has also been used to assess response to treatment. RECIST 1.1 represents a further update, taking into account new concepts such as revised minimum dimensions for lymph nodes and an approach to lesions that become non-measurable. Based on experience and published literature, the hypothesis of merging the 2 above-mentioned criteria in mRECIST 1.1 for mesothelioma and the use of iRECIST for the application to immune-based therapies (iRECIST) was considered. **Purpose:** Support the importance of studying pleural mesothelioma in a reliable and reproducible way, through a scrupulous methodology, applying the mRECIST1.1 and iRECIST criteria. **Conclusions:** Adoption of a standardized study metodology can make the study of PM reproducible and correct.

## Introduction

Malignant pleural mesothelioma is the most common primary tumor of the pleura and has a strong association with occupational exposure to asbestos.^1^ The application of conventional response criteria for the study of malignant mesothelioma has always been difficult due to its unique growth pattern. Typically, the growth pattern of malignant mesothelioma could be attributed to a “cortex” around the pleural surface, so in computed tomography (CT) scan it may not appear as the usual spherical lesions with diameters measurable in 2 dimensions. WHO proposes criteria that are not very suitable for response assessment in malignant mesothelioma, since they were constructed to assess the type of disease measurable only in 2 dimensions.^1^For a more appropriate assessment, RECIST (Response Evaluation Criteria in Solid Tumors) should be used.^2^However, it should be kept in mind that the choice of measurement sites in mesothelioma is not an easy thing, it requires good knowledge and practice of the measurement method.^3^

**Figure 1.**
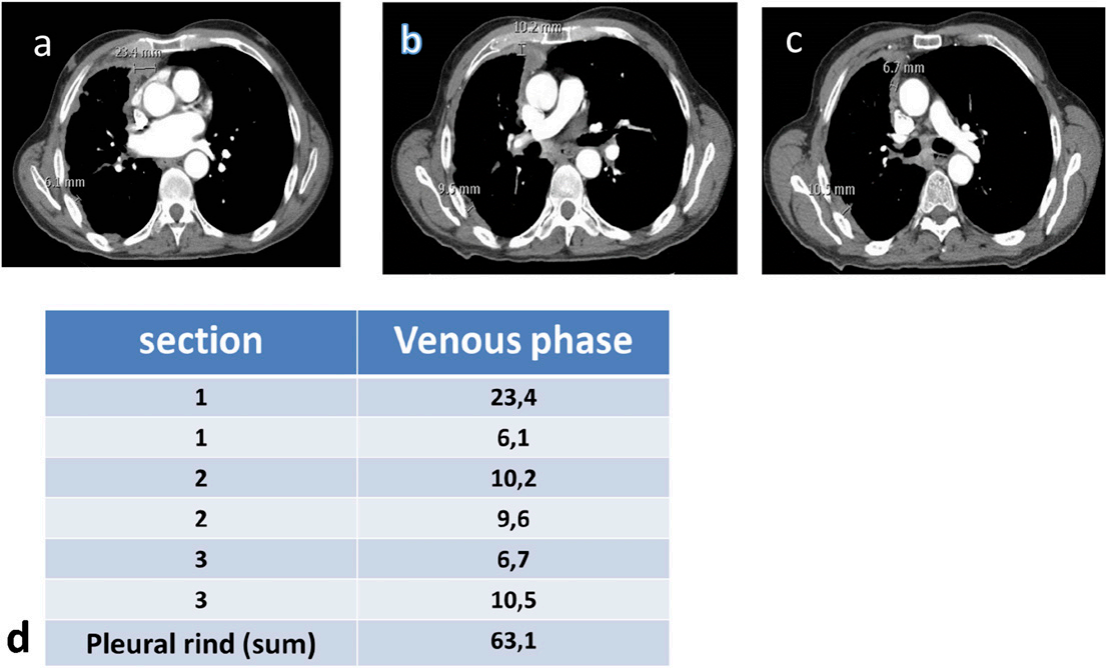
Example of measurement of detectable lesions obtained by measuring the thickness of the tumor perpendicular to the chest wall. Pleural tumor thickness was measured at 2 different locations on each of 3 levels selected for unidimensionally measurable lesions (a, b, and c). Subsequently, a total measurement was calculated by adding the 6 measurements, thus obtaining the sum of the pleural crust (d).

The modified RECIST Response Evaluation Criteria (mRECIST) for mesothelioma was introduced to overcome the limitations of RECIST. In detail, mRECIST measures tumor thickness perpendicular to the chest wall or mediastinum, reducing the number of pleural sites measured to 6.^2,3^ RECIST 1.1 represents a further update, taking into account new concepts such as revised minimal lymph node size and assessment of lesions that become non-measurable. Advantages would include measurement of minimally measurable disease, definition of measurable lesions, assessment of non-pleural pleural disease, assessment of pathological lymph nodes, progressive disease, and inclusion of bilateral pleural disease.^4^ mRECIST took a different approach by selecting 6 measurement sites “in 2 locations at 3 separate levels on axial CT scan slices at least 1 cm apart, trying to correlate them to anatomical landmarks in the chest to obtain a reproducible assessment over time^3,4^ (Figure 1(a)-(d)). For all these reasons, based on experience and published literature, the hypothesis of merging the 2 above-mentioned criteria in mRECIST 1.1 for mesothelioma has been considered. The introduction of mRECIST 1.1 recommends reducing the minimally measurable disease for mesothelioma from 10 mm to 7 mm and subsequently measuring at follow-up all sites that shrink below the minimally measurable dimension (as per RECIST 1.1) and a default value of 2 mm (not compliant with RECIST 1.1) in case the tumor is too thin to be measured.^4,5^mRECIST 1.1 promotes the adoption of iRECIST for immunotherapy clinical trials, necessarily having to consider pseudoprogression and delayed response [2-3-6]. The aim of our review is to highlight the importance of studying PM in a reliable and reproducible way by carefully applying the mRECIST 1.1 and iRECIST criteria.

### Imaging CT

**Figure 2.**
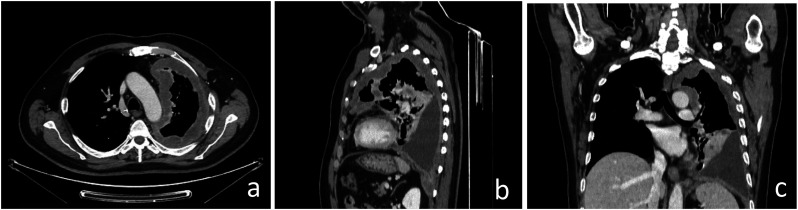
Example of malignant pleural mesothelioma studied with multidetector CT images in 3 planes with high spatial resolution (slice thickness of approximately 1-2 mm in the axial plane (a) and 2-3 mm in the sagittal and coronal planes (b and c).

**Figure 3.**
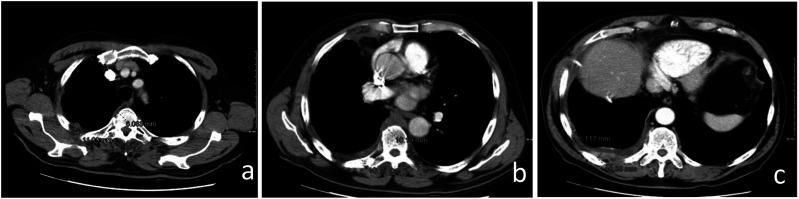
Example of measurements of pleural mesothelioma, present in sites consistent with clinical relevance, up to a maximum of 6 in no more than 3 sections at least 1 cm apart, preferably above the left atrium (a) and below the aortic arch (b and c) to try to standardize reproducibility.

**Figure 4.**
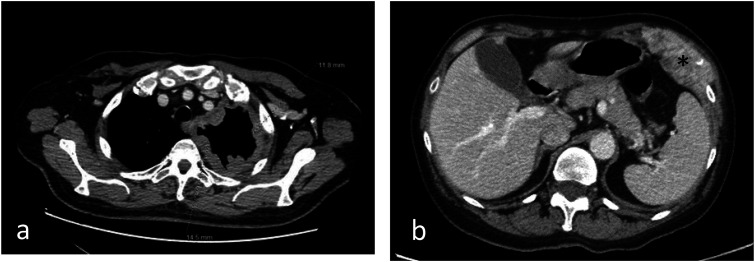
Example of advanced disease characterized by left circumferential pleural thickening, ipsilateral lung volume loss and “crusted” infiltration of the lung parenchyma (a) and chest wall infiltration (*) (b).

**Figure 5.**
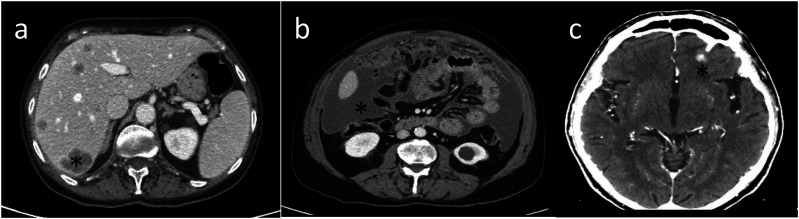
Example of extrathoracic extension of the disease, with hepatic invasion (*) (a), peritoneal carcinomatosis (*) (b), and brain metastases (*) (c).

**Figure 6.**
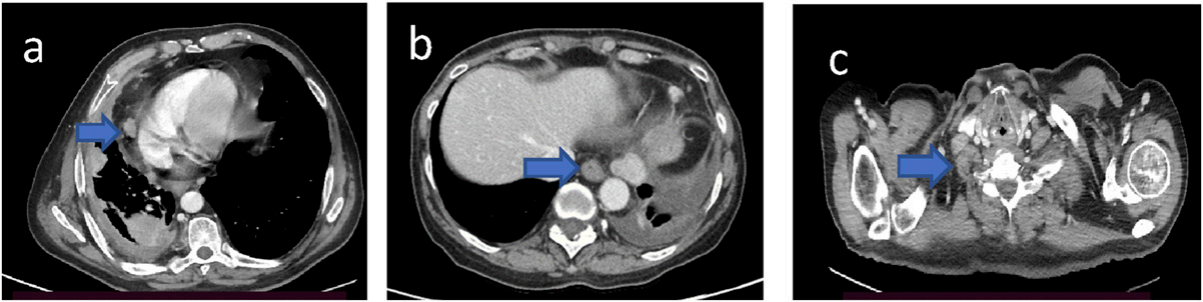
Mediastinal (a), para-aortic (b), and laterocervical (c) lymph node involvement, with short axis of 10 mm or more, considered pathological.

Multidetector computed tomography (CT) is the technique of choice for the morphological assessment of PM and the primary tool for staging and treatment planning. Modern CT scanner technology allows the acquisition of images that can reliably reproduce the thoracic and abdominal anatomy of interest in any imaging plane, favoring the study of extremely thin and difficult-to-detect findings and structures (such as the diaphragm, pericardium or peritoneal surfaces).^5,6^The iMIG Pleural Imaging Expert Panel recommends interpreting multidetector CT images in 3 planes with high spatial resolution (section thickness of approximately 1-2 mm in the axial plane and 2-3 mm in the sagittal and coronal planes), precisely because PM shows multiplanar development and growth and is characterized by discontinuous lesions, almost always non-spherical in shape on multiple CT slices (Figure 1). Pleural measurements are taken at sites consistent with clinical relevance (maximum 6 in no more than 3 sections at least 1 cm apart), preferably over the left atrium and under the aortic arch to try to standardize reproducibility^4^ (Figure 2). To achieve optimal target organ coverage, the scan volume should include the entire thorax with a caudal extension to the level of L3. Intravenous administration of iodinated contrast medium is essential to achieve an increase in lesion enhancement that significantly improves CT detection and quantification of PM deposits.^4,6^ Pleural thickening involving the mediastinal, parietal, and/or scixophilic pleura in a nodular, lobular, or circumferential pattern on a contrast-enhanced CT scan greater than 1 cm is highly suggestive of PM. Pleural plaques and effusions may occur in approximately 75%-20% of patients, respectively. Circumferential pleural thickening, ipsilateral lung volume loss, and crusting of the lung parenchyma suggest frankly advanced disease (Figure 3(a)). In case of contralateral pleural involvement, transdiaphragmatic extension, presence of multifocal metastases, spread to the spine, pericardium, mediastinal tissues, or chest wall, the disease is defined as unresectable (Figure 3(b)). Signs of chest wall invasion include bone damage, rib displacement, infiltration of intercostal muscles, and obliteration of typical extrapleural fat layers (Figure 4). Examples of extrathoracic extension of the disease are shown in Figure 5. Lymph node enlargement itself does not demonstrate metastatic nodal dissemination, so its accuracy is still not ideal.^5,7^ In case mediastinal lymph nodes have a short axis of 10 mm or more, they will be considered pathological based on specific CT morphological criteria (Figure 6).

## Discussion

The modified RECIST criteria are intended to explicitly address the distinct developmental pattern associated with malignant pleural mesothelioma, often addressing difficult and unclear circumstances that have arisen during the interpretation of responses. Although the effectiveness of using mRECIST 1.1 compared to the previous mRECIST and RECIST 1.1 systems has brought important improvements in the effective quantification of tumor burden and assessment of treatment response, it is important to consider that only the adoption by radiologists of a standardized study methodology can make the study of PM reproducible and correct. The creation of an automated measurement format could improve the speed and reproducibility of measurement^4^ and help to mitigate the possible problem of interobserver variability. Performing measurements with the same observer or a control group can prevent this problem.^7,8^A group of physicians with limited expertise in measuring mesothelioma in clinical trials should be subject to rigorous criteria to verify their applicability. Immunotherapy is currently undergoing intensive clinical trials in mesothelioma and benefits have been reported in this setting. Clinical trials of these agents are accompanied by challenges in assessing tumor response primarily due to immune-related pseudoprogression, where increased peritumoral inflammation may be visually indistinguishable from the tumor itself, resulting in perceived tumor mass on CT. Continuous simultaneous collection of RECIST 1.1 measurements (along with iRECIST measurements) is encouraged for clinical trials. iRECIST is expected to improve the identification of atypical responses, including delayed responses after pseudoprogression. iRECIST allows patients to continue in clinical trials in the event of initial progression of baseline target lesions or evidence of new lesions, introducing the concepts of unconfirmed progressive disease and confirmed progressive disease. It should also always be remembered that good medical practices require that all CT studies must always be optimised, remembering the fundamental principles of radiation protection^8^ and through decisions shared with different specialists in the sector, through teamwork with a multidisciplinary approach.^9^

## Conclusion

Specific guidelines for the study and evaluation of PM treatment response should include the principles of mRECIST 1.1 together with the principles of iRECIST applied to progressive disease. It is essential to consider that only the adoption of a standardized study methodology can make the study of PM reproducible and correct. iRECIST should improve the identification of atypical responses, such as delayed responses after pseudoprogression.
